# To VBAC or Not to VBAC

**DOI:** 10.1371/journal.pmed.1001191

**Published:** 2012-03-13

**Authors:** Catherine Y. Spong

**Affiliations:** National Institute of Child Health and Human Development, Bethesda, Maryland, United States

## Abstract

Catherine Spong discusses new research in *PLoS Medicine* that sheds more light on the risks of uterine rupture for women attempting a trial of labor following previous cesarean section.

**Linked Research Articles**

This Perspective discusses the following new studies published in *PLoS Medicine*:

Fitzpatrick K, Kurinczuk JJ, Alfirevic Z, Spark P, Brocklehurst P, et al. (2012) Uterine Rupture by Intended Mode of Delivery in the UK: A National Case-Control Study. PLoS Med 9(3): e1001184. doi:10.1371/journal.pmed.1001184

A case-control study using UK data estimates the risk of uterine rupture in subsequent deliveries amongst women who have had a previous caesarean section.

Crowther CA, Dodd JM, Hiller JE, Haslam RR, Robinson JS, et al. (2012) Planned Vaginal Birth or Elective Repeat Caesarean: Patient Preference Restricted Cohort with Nested Randomised Trial. PLoS Med 9(3): e1001192. doi:10.1371/journal.pmed.1001192

A study conducted in Australia provides new data on the outcomes for mother and baby associated with either planned vaginal birth, or elective repeat caesarean section following a previous caesarean section.

Pregnancy can be associated with significant risks for the mother and baby regardless of the presence of medical or obstetrical complications. In the presence of complications such as hypertension, multiple gestation, or prior cesarean delivery, the risks to the health of the mother and baby increase. Numerous studies, including two published in *PLoS Medicine* recently [1],[2], have shown that risks such as uterine rupture are higher for women attempting a trial of labor following a previous cesarean delivery than those having an elective repeat cesarean delivery; however, the overall risks are low in both groups.

## Uterine Rupture

Rupture at the site of the prior cesarean scar is the most feared complication of trial of labor in women with a prior cesarean delivery. While rare, its consequences to mother and baby are dire. Evidence provided by these two new articles helps improve our understanding of the risks and consequences of uterine rupture. In the first article, Kathryn Fitzpatrick and colleagues report on a population-based case control study in the United Kingdom of 159 women with uterine rupture occurring both at term and preterm [1]. In the second, Caroline Crowther and colleagues report predominantly on a patient preference cohort study of 2,323 Australian women with single prior cesarean and current singleton cephalic gestation at 37+ weeks eligible for vaginal birth after cesarean (VBAC), of whom 1,225 planned VBAC and 1,098 planned an elective repeat cesarean [2]. In both studies, the estimated incidence of uterine rupture was approximately 1 in 500 women planning VBAC and 1 in 1,000 women planning an elective repeat cesarean. These numbers are somewhat lower than what has been previously reported and what is currently quoted to patients, which is somewhat encouraging.

## Neonatal Outcome

Crowther and colleagues also reported on neonatal outcomes and found that the risks of fetal or infant death, or serious adverse infant outcomes, were significantly lower in planned elective repeat cesarean (0.9%) versus the planned VBAC group (2.4%). These two studies provide important information previously missing in the literature, enabling an assessment of risks based on a woman's “intent” of delivery method. To date, most of the literature has been based on the numbers utilizing the actual delivery method, not the intent. The literature based on actual delivery method, not intent, makes counseling difficult, as women may intend to have an elective repeat cesarean, but may go into labor and either have an indicated cesarean or a vaginal birth. Counseling the women near term using data derived from actual delivery methods, then, is not accurate. Both of these studies have captured this variable of intent, which is clearly important: in the Crowther et al. study, nearly 98% of women who planned an elective repeat cesarean succeeded, yet only 57% of those who planned a vaginal birth after cesarean did. It is also interesting that almost a quarter of the women in the planned VBAC group had an elective repeat cesarean, presumably because they changed their minds or it became indicated to proceed with cesarean.

## What Are the Limitations of These Two New Studies?

Included in the Crowther study was an attempt at obtaining definitive data on the subject using the gold-standard, randomized controlled trial (RCT). Although this is the ideal method to answer the question about neonatal risks without the bias that comes from non-randomized designs, clearly it was a difficult trial to recruit into, as only 22 patients were recruited. Indeed, it is unlikely that such an RCT will ever be easy or even possible to complete. Delivery is a very personal time for patients and families. Many events surround both the history of the initial cesarean and the timing of the subsequent delivery that may hinder acceptance of randomization in this setting. In addition, the accepted ability for patients to select their delivery method, with some patients electively choosing cesarean as a first delivery method, makes randomization even more difficult. It is also challenging to study the most dreaded outcome, uterine rupture, because it is so rare. Therein lies the need for retrospective case control studies such as the one by Fitzpatrick and colleagues. The most important concern with case-control studies is that they may not have controlled for all confounders. The decision, or intent, for one route of delivery versus another may have been affected by maternal or fetal factors that may also have affected the outcome. However, any such factor may have biased results toward a worse outcome for the repeat cesarean.

## What Are the Risks for Women and Their Babies?

One of the frequently overlooked risks of repeat cesarean versus trial of labor involves the timing of delivery. While awaiting the onset of labor, the woman who has elected a VBAC may experience a maternal or fetal complication that could have been prevented had she elected and undergone a repeat cesarean. Examples of such complications include preeclampsia and stillbirth. In the Crowther study, there were two antepartum unexplained stillbirths, both in the intended VBAC group, with an overall rate within the anticipated rate of stillbirth at term. It is important to recognize that a higher rate of stillbirth in the intended VBAC group cannot be considered a result of intended mode of delivery, but more likely a result of length of gestation. Thus, it appears to be the timing of the delivery that affects the stillbirth rate, not the mode of delivery. In the Crowther study, cesarean deliveries were performed between 38 and 40 weeks of gestation; the average gestational age at delivery was 38.8 weeks in the planned elective repeat cesarean group and 40.0 weeks in the planned VBAC group. Thus, the women planning VBAC remained pregnant over a week longer than those having a cesarean. It is not possible using these data to determine whether planned mode of delivery prevents stillbirth—that would require both groups to deliver at approximately the same gestational age.

Another frequently under-discussed risk is that of the impact of multiple cesareans on both the mother's and future children's health. Numerous studies have shown multiple cesareans increase the risk of the placenta implanting abnormally (placenta previa with or without placenta accreta) that can result in significant hemorrhage or hysterectomy and may be life-threatening to both the mother and baby.

## Putting the Risk in Perspective

It is well accepted that pregnancy is associated with risks, both to the mother and to the baby. However, as we quantify risks for patients, some are discussed more actively with patients than others, perhaps unintentionally magnifying them out of proportion with the actual risk. For women with a prior cesarean, the risk of uterine rupture, as found in these studies, is between 0.1% and 0.2%. Although these two articles do not provide sufficient data to evaluate rupture-related fetal loss rates, previous work indicates a 6% perinatal death rate from uterine rupture [3]; thus, the perinatal loss rate from uterine rupture is 0.006%. If we compare this to other pregnancy complications, it is a generally accepted level of risk. In the largest study evaluating midtrimester amniocentesis, fetal loss following midtrimester amniocentesis is 0.1% [4], the risk of fetal or neonatal death associated with cord prolapse is 0.01%–0.1% [5], perinatal mortality due to placental abruption is 0.1% [6], and the risk of infant death due to sudden infant death syndrome is 0.1% [7]. Although these complications are all rare, and the perinatal loss risk is far less than many other accepted obstetrical risks, the concern over uterine rupture at VBAC has numerous extra precautions built in, to the extent of limiting access to this delivery option. The reason may be related to the fact that a physician performed the initial cesarean, thus elevating the concerns of uterine rupture over other comparably risky, but spontaneous complications.

Everything one does is associated with risk, from walking to the store, to driving a car, to the foods one consumes. Some of these risks we elevate to have major impact in our decisions even if that risk is remote, whereas other more frequent risks we easily accept and move forward. Given the major complications associated with multiple cesareans, to both mother and baby, women should carefully evaluate the immediate risks in the current pregnancy with the longer-term risks of multiple cesareans. Despite the low overall risks for attempting a vaginal delivery after cesarean, in many areas, women do not have the opportunity to choose their preferred method of delivery, as VBAC may not be offered by her hospital, her physician, or, where relevant, her health care insurance plan.

## How Will These Studies Affect Practice?

Unfortunately, the discussion regarding the minimal risk associated with VBAC, and the need to advocate for more VBAC, is likely to remain academic. Moreover, these two studies are likely to increase the fear of trial of labor rather than alleviate it. When faced with a 2.4% relative risk of perinatal death or serious adverse outcome with an intent for VBAC versus a 0.9% relative risk for an intent for repeat cesarean, mothers are unlikely to choose to await labor regardless of the counseling or the risk to the mother.

What is needed is a new way to counsel patients, be it with new data or better categorization of patients' risk. The study by Fitzpatrick and colleagues confirms that oxytocin use increases the rates of uterine rupture. Most clinicians now shy away from oxytocin use, particularly for labor induction. Grobman et al. have shown that the risk and success of VBAC can be better categorized using a validated prediction model [8]. The risk is not static, but changes during pregnancy and intrapartum as the patient condition changes and new factors develop.

In the end, the onus falls on the clinicians. All this discussion would be moot and neither the patient nor the clinician would have to fret about whether to attempt a trial of labor or choose a repeat cesarean if the first cesarean had been prevented.

## Abbreviations

VBAC: vaginal birth after cesarean
